# Yellow Fever, Considered in Its Historical, Pathological, Etiological and Therapeutical Relations

**Published:** 1856-09

**Authors:** 


*Yellow Fever, considered in its historical, pathological, etiological and therapeutical relations.* Including a sketch of the disease as it has occurred in Philadelphia from 1699 to 1854. With an examination of the connections between it and the fevers known under the same name in other parts of temperate, as well as in tropical regions. By R. La Roche, M. D., Member of the American Philosophical Society, etc., etc., etc. In two volumes. Philadelphia: Blanchard & Lea. 1855.

This truly great work has already been noticed in the Buffalo Medical Journal, and has received commendation at the hands of all the American medical journalists. In again calling attention to it; we are actuated by a desire that a work of such magnitude and importance shall not be tardily or unduly appreciated by the medical profession of this country. Highly prized as it will be in all future time, we hope that a proper estimation of the author's labors may be manifested during his life time and not remain in abeyance as merely an honorable legacy to his descendants. In saying this we may add, that we have not the pleasure of even a personal acquaintance with the author — the remark is prompted solely by the feelings produced by a pretty thorough examination of the two large volumes before us.

To have gathered together, perused, collated, composed, and, after careful analysis, selected, condensed and arranged all that is worthy of preservation in the host of publications, in different languages, pertaining to yellow fever, was an undertaking which may be justly styled peculiar. Some idea may be formed of the task by a glance at the bibliographical list which the author gives, covering in small print, forty-five pages! But the time and industry, to say nothing of talent and erudition, which the execution of the undertaking demanded, will be best understood by those who have had some experience in labors of a similar character. Moreover, the task of preparing these volumes could not have been enlivened by the interest which attaches in a new field of scientific exploration; but, dealing so extensively with facts and opinions contained in the literature of the subject, the drudgery of the occupation would have been intolerable save for the animating motives derived from a sense of its usefulness.

Of the value of the work one is prepared to form an adequate estimate after availing himself of it, in order to be prepared to discuss the various questions connected with yellow fever, without having had opportunities for the clinical study of the disease. To consult the scattered fragmentary publications (*disjecta membra*) which constitute the literature of the subject, a large proportion of which is inaccessible to the majority of medical readers, would require time and patience more than most persons could bestow on this object. It is now not only unnecessary, but hardly a desideratum, even to those who wish to investigate these questions the most fully.

Dr. La Roche's work embodies all that is wanted. It is a compendium of the whole vast literature of yellow fever. Thanks to his labors, the medical scholar who desires to be profoundly conversant with all that pertains to the subject need not go beyond these two portly volumes. As embodying whatever is important in all that has been hitherto written on the subject, it will be a work for reference not less valuable in ages to come than now. Its merit, however, by no means consists solely in its completeness as an encyclopædian work. The author presents the conclusions to which he is led by a philosophical investigation of the facts and opinions gathered from past and contemporaneous publications. Of the soundness of the conclusions the reader can judge from the data which are spread before him.

An analytical review of a work of the size and character of this, would be both undesirable and, within proper limits, impracticable. Our object, as already intimated, in writing this brief article, is to commend it to the attention of those who take an interest in the progress of American medical literature, and to express our admiration of the generous, noble spirit of the distinguished author, who has devoted years of tedious toil in the execution of a task which, intrinsically, offered few if any attractions; which could not be expected to create a sensation such as is produced by novel and striking developments, gratifying for a time an author's self-love, and which held out no inducements of pecuniary reward. The pleasures incident to useful occupation, however laborious, and a desire to render a solid, enduring service to the medical profession and to mankind, could alone have prompted and secured the performance of such a task. Let the author be gratified by the reflection that his labors are appreciated, and as conducive to this end let it be considered that the slightest acknowledgment which can be made of the honor which the publication of this work reflects on our national medical literature as well as on the author, is for every enlightened practitioner to give it a place in his library. A. F.

